# The cost of illness attributable to diabetic foot and cost-effectiveness of secondary prevention in Peru

**DOI:** 10.1186/s12913-015-1141-4

**Published:** 2015-10-26

**Authors:** María Kathia Cárdenas, Andrew J. Mirelman, Cooper J. Galvin, María Lazo-Porras, Miguel Pinto, J. Jaime Miranda, Robert H. Gilman

**Affiliations:** CRONICAS Centre of Excellence in Chronic Diseases, Universidad Peruana Cayetano Heredia, Av. Armendariz 497, Lima 18, Miraflores, Peru; Centre for Health Economics, University of York, York, UK; Stanford University, Stanford, CA USA; Unidad de Conocimiento y Evidencia, Universidad Peruana Cayetano Heredia, Lima, Peru; Servicio de Endocrinología, Hospital Nacional Cayetano Heredia, Lima, Peru; Facultad de Medicina “Alberto Hurtado”, Universidad Peruana Cayetano Heredia, Lima, Peru; Department of International Health, Johns Hopkins Bloomberg School of Public Health, Baltimore, MD USA

**Keywords:** Chronic diseases, Cost analysis, Cost effectiveness analysis, Developing countries, Diabetic foot, Diabetes mellitus

## Abstract

**Background:**

Diabetes mellitus is a public health challenge worldwide, and roughly 25 % of patients with diabetes in developing countries will develop at least one foot ulcer during their lifetime. The gravest outcome of an ulcerated foot is amputation, leading to premature death and larger economic costs.

**Methods:**

This study aimed to estimate the economic costs of diabetic foot in high-risk patients in Peru in 2012 and to model the cost-effectiveness of a year-long preventive strategy for foot ulceration including: sub-optimal care (baseline), standard care as recommended by the International Diabetes Federation, and standard care plus daily self-monitoring of foot temperature. A decision tree model using a population prevalence-based approach was used to calculate the costs and the incremental cost-effectiveness ratio (ICER). Outcome measures were deaths and major amputations, uncertainty was tested with a one-way sensitivity analysis.

**Results:**

The direct costs for prevention and management with sub-optimal care for high-risk diabetics is around US$74.5 million dollars in a single year, which decreases to US$71.8 million for standard care and increases to US$96.8 million for standard care plus temperature monitoring. The implementation of a standard care strategy would avert 791 deaths and is cost-saving in comparison to sub-optimal care. For standard care plus temperature monitoring compared to sub-optimal care the ICER rises to US$16,124 per death averted and averts 1,385 deaths.

**Conclusion:**

Diabetic foot complications are highly costly and largely preventable in Peru. The implementation of a standard care strategy would lead to net savings and avert deaths over a one-year period. More intensive prevention strategies such as incorporating temperature monitoring may also be cost-effective.

**Electronic supplementary material:**

The online version of this article (doi:10.1186/s12913-015-1141-4) contains supplementary material, which is available to authorized users.

## Background

Diabetes mellitus is a chronic disease that presents a challenge for health care systems worldwide. One major complication of diabetes mellitus is the development of diabetic foot [1, 2], which may be costly for families, health systems and society. In developing countries 25 % of diabetic patients develop at least one foot ulcer during their lifetime [3, 4] as a result of delays in diabetes diagnosis, poor access to health care and poor treatment adherence [5].

Diabetic foot is characterized by retardation and death of peripheral nervous tissue in the foot, which results in susceptibility to ulcerative infection. Over time, the gravest outcome from ulcerated feet is amputation, which may also be accompanied by pain, disability, risk of depression and decreased quality of life [3].

In 2005, the International Diabetes Federation (IDF) recommended yearly foot check-ups for all patients with diabetes in order to identify those at high-risk of ulceration [6]. In Peru, 30 % of people with diabetes were unaware of their condition [7] and only 8 % of patients undergoing treatment met control targets [7].

The objective of this analysis is to estimate the cost-of-illness (COI) attributable to diabetic foot in the patients at high-risk of ulceration in Peru for different secondary prevention strategies. A cost-effectiveness analysis is also conducted. We consider three strategies: sub-optimal care or usual care in Peru (used as comparator), standard care based on IDF recommendations, and an intensive strategy based on standard care plus temperature monitoring.

## Methods

**Fig. 1:**
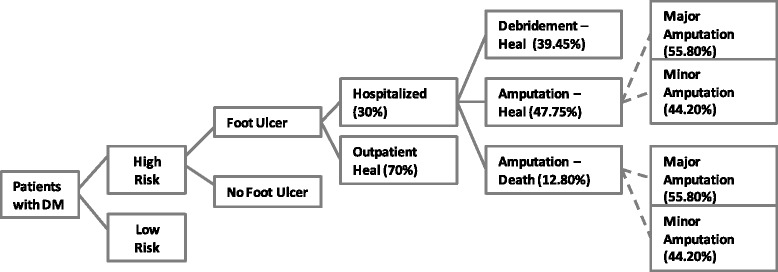
Decision tree diagram for patients at high-risk of ulceration, diabetic foot and related outcomes

i) Design
  A COI study was developed following an ingredients-based approach where unit costs for resources are multiplied by the quantity used. A decision tree model (see Fig. 1) was constructed to show cases of patients at high-risk of ulceration, diabetic foot and related outcomes. A population prevalence-based approach was performed to illustrate the costs and benefits that can be achieved in the first year of each strategy. All sources for key parameters are listed in Table 1.

**Table 1:** Epidemiology and cost inputs

Parameter	Value	Lower estimate	Upper estimate	Reference
Epidemiology				
a. Total population with Type 2 diabetes mellitus	942,000			[11]
b. % diabetics at high risk for ulcer	21.90 %			[12, 13]
c. Prevalence of ulcer in those at high-risk	22.18 %	15.53 %	28.84 %	[9, 14, 15] Own assumption: values for lower and upper estimate +/−30 %
c1. Effectiveness (reduced prevalence) with standard care	45.00 %	30.00 %	60.00 %	[9, 14, 15] Own assumption: assumed values for lower and upper estimate.
c2. Effectiveness (reduced prevalence) with standard care plus temperature monitoring	78.81 %	65.00 %	85.00 %	[9, 14, 15] Own assumption: assumed values for lower and upper estimate.
d. % with ulcer receiving hospital care	30.00 %	21.00 %	39.00 %	[16] Own assumption: values for lower and upper estimate +/−30 %
e. % with ulcer receiving outpatient wound management	70.00 %	61.00 %	79.00 %	[16] Own assumption: values for lower and upper estimate.
f. % at hospital with amputation and heal	47.75 %	33.43 %	62.08 %	[16] Own assumption: values for lower and upper estimate +/−30 %
f1. % with major amputation	55.80 %			[16]
f2. % with minor amputation	44.20 %			[16]
g. % at hospital with debridement and heal	39.45 %	25.12 %	53.77 %	[16] Own assumption: values for lower and upper estimate +/−30 %
h. % at hospital that die of diabetic foot after an amputation	12.80 %			[16]
Direct costs (in 2012 US dollars)				
Prevention				
Sub-optimal care	65	45	84	[17, 18] Other local prices. Own assumption: values for lower and upper estimate +/−30 %
Standard care	185	129	240	[17, 18] Other local prices. Own assumption: values for lower and upper estimate +/−30 %
Standard care plus temperature monitoring	406	285	528	[17, 18] Other local prices. Own assumption: values for lower and upper estimate +/−30 %
Treatment				
Wound management without hospitalization	79			[17, 18] Other local prices.
Debridement	1,022			[17, 18] Other local prices.
Minor amputation	5,153			[17, 18] Other local prices.
Major amputation	7,360	5,152	9,568	[17, 18] Other local prices. Own assumption: values for lower and upper estimate +/−30 %
Indirect costs (in 2012 US dollars)				
Productivity loss from premature death	6,719			Peru's basic salary in year 2012; 3 % discount rate.
ii) Definition of prevention strategies
  The sub-optimal care or baseline situation was assumed to include an annual visit to a physician and podiatrist where routine tests are performed once, but neither education nor appropriate footwear is provided.
  The second strategy, called standard care, was based on IDF guidelines [6], which emphasizes attention by a cadre of medical professionals, bi-monthly consultations, a higher frequency of tests, podiatrist consultation with foot evaluation, education and use of insoles (see Table 2).

**Table 2:** Major assumptions in cost-of-illness estimation

A. Wound management without hospitalization
Outpatient: 1 first visit, 2 visits of control, 3 minor healing procedures in a hospital. Test: HbA1C, lipid profile, X-ray. Medication: Clindamycin 300 mg qid for 2 weeks.
B. Debridement
Inpatient: Emergency consultation, 6 days of hospitalization, evaluation by anesthesiologist and cardiologist, anesthesiology medication and surgical materials, debridement procedure, intermediate care unit and 6 wound healing procedures. Test: Pre-surgery tests, antibiogram, HbA1C, lipid profile and X-ray. Medication: Intravenous antibiotic (Ampicillin/Sulbactam 1.5 g qid for 3 days), oral antibiotics for 11 days and peripheral line. Outpatient: Consultations with physician until healed at the hospital and materials for dressing changes.
C. Amputation
Inpatient: Emergency consultation, 10 days (minor amputation) or 19 days (major amputation) of hospitalization, evaluation by anesthesiologist and cardiologist, anesthesiology medication and surgical materials, amputation procedure, intermediate care unit and blood transfusion. Test: Pre-surgery tests, bacteriology study, HbA1C, lipid profile, white cells count, X-ray, Doppler echography, arteriography, MRI, tissue biopsy. Medication: Intravenous antibiotic (Ampicillin/Sulbactam 1.5 g qid for 3 days in minor amputation and 5 days in major amputation), oral antibiotics (11 days in minor and 16 days in major amputation) and peripheral line. Outpatient: Consultations with physician and podiatrist until healed, materials for dressing changes (assuming that a nurse or a trained person at home is in charge of this procedure). Others: Rehabilitation sessions (40 for minor amputation and 50 for major amputation), orthopedic supplies for foot amputation (crutches and orthopedic foot) or for leg amputation (crutches, orthopedic leg, wheelchair), caregiver at home (conservative assumption of 6 months at Peru´s basic salary or 12 months working partial time).
D. Premature death
We assumed that 2 years (retirement age of 65) of paid productive work were lost due to the death and discounted at an annual rate of 3 %. Minimum wage rate in Peru amounts to PEN 750 in year 2012 (equivalent to US$284). We assumed a monthly income equal to minimum wage. The estimated indirect cost was US$6,719, which is the net value of the lost earnings for the next 2 years.
E. Sub-optimal care
Outpatient: 1 annual consultation with physician and podiatrist. Test: 1 annual testing of HbA1C, lipid profile, creatinine, electrocardiogram, X-ray.
F. Standard care
Outpatient: 6 consultations with physicians, 1 consultation with the podiatrist and 1 education session with a nurse. Test: 3 annual evaluations of HbA1C, 1 annual testing of lipid profile, 2 creatinine tests, 2 electrodiagrams and 1 X-ray. Others: protective footwear (a pair).
G. Standard care plus temperature monitoring
Similar to standard care, but in addition: thermometer and daily phone calls assisted by a nurse or a trained person (about 5 minutes per patient everyday).

*HbA1C* glycosylated hemoglobin, *MRI* magnetic resonance imaging
  Thirdly, a standard care plus temperature monitoring strategy was included, which added daily self-monitoring of foot temperature to the standard care. This involves the use of a handheld thermometer for recording and monitoring fluctuations in foot temperature of individuals. If patients find a high degree of fluctuation in temperature between both feet, higher or equal to 2*.*2 °C, they need to call a nurse and schedule a consultation. This strategy has been applied in a number of clinical trials yielding consistent positive results [8–10].
iii) Epidemiology
  Our calculations considered the Peruvian adult population (18–79 years old) with Type 2 diabetes mellitus who were at high-risk of developing diabetic foot ulcer due to severe neuropathy with foot deformity or a history of ulceration. We used the IDF national data for the prevalence of Type 2 diabetes in Peru, estimated at 5*.*38 % in year 2011 [11]. This resulted in nearly 942,000 people having the disease. The prevalence of being at high-risk for diabetic foot was defined as the prevalence rate of severe neuropathy among diabetic patients, assumed to be 21*.*9 % based on previous studies for developing countries [12, 13]. It was calculated that the population in Peru with severe neuropathy was 206,298 persons. We have excluded patients with peripheral ischemia from our approach given that this population was also excluded in clinical trial studies exploring temperature monitoring as a preventative strategy for diabetic foot [8–10].
  The three different prevention strategies affected a reduction in the one-year ulceration rate for those that were at high-risk for diabetic foot. To illustrate the achievable health benefits, it was assumed that each strategy was introduced at scale. For the sub-optimal care strategy as baseline, the ulceration prevalence rate was assumed to be 22*.*18 %. This figure came from considering the ulceration prevalence rate for the standard care in a clinical trial study reported as 12.2 % [9], together with an effectiveness estimations of 45 % derived from the United States Center for Disease Control and Prevention (CDC) [14, 15] when moving from a scenario of sub-optimal care to standard care. An even higher reduction of 79 % in the baseline rate of ulceration is achieved with the standard care plus temperature monitoring, achieving a 4.7 % of ulceration rate [9].
iv) Clinical outcomes
  We determined the likely clinical outcomes for different scenarios. The data was for a one-year period related to diabetic foot treatment and complications such as ulcer development, wound management, debridement procedure, amputation and death.
  We considered the parameters from a Brazilian study [16] that provides rates for hospitalization and outcomes attributed to diabetic foot ulceration in patients attended by the public health system. This kind of study has not been implemented in Peru and there is a lack of reliable epidemiological data from the health statistics at hospitals. In the Brazilian COI study [16], 70 % of ulcerated patients heal without hospitalization, and therefore, we assumed that those patients only receive outpatient wound management. The other 30 % of patients require hospitalization, with 39.5 % healing with primary care (assumed as debridement procedure), 47.8 % healing by a minor or major amputation, and 12.8 % dying after amputation surgery. Our assumption here is that all patients who die had an amputation in the same year. Among those patients who undertook amputation surgery in the Brazilian study, 44.2 % received a minor amputation and 55.8 % a major amputation. Table 1 shows the values used and assumptions made for this study.
v) Cost data
  Direct costs of resources used to prevent or manage the disease were registered under a societal perspective. We identified the main medical procedures that can be carried out in Peru related to each stage of diabetic foot. We followed an ingredients-based approach for our costing analysis (see Additional file 1). We estimated the number of specific procedures, personnel, medical supplies, examinations and medications for each disease stage according to the IDF guidelines. The unit costs of treatment were taken from a list of tariffs in a public hospital of the Ministry of Health in Peru [17]. We considered generic drug prices [18].
  Costs were registered at the local currency *Peruvian Nuevos Soles* (PEN) and converted to US dollars (US$), considering an average exchange rate of 2*.*64 PEN per dollar in year 2012 [19].
  Additionally, the indirect costs due to premature death were measured using a human capital approach. Minimum wage rate in Peru amounts to US$284 in year 2012. We assumed a monthly income equal to minimum wage and a mean age of 63 years for premature mortality, according to the primary data of patients with neuropathy in a public hospital in Lima [12]. We considered that two years, with a retirement age of 65, of paid productive work were lost due to the death and discounted at an annual rate of 3 %.
vi) Cost-effectiveness analysis
  Cost-effectiveness is examined in terms of cost per deaths averted. The analysis consisted in estimating the incremental cost-effectiveness ratio (ICER) by estimating the additional costs and deaths averted for each of the preventive strategies compared to the sub-optimal strategy. Since the analysis only looked at costs and effects for one-year, there was no discounting.
vii) Sensitivity analysis
  We conducted multiple one-way sensitivity analysis to assess the uncertainty of the key input parameters. The variables of interest were independently varied according to their plausible range from Table 1. Most of the variables were varied using a range of +/− 30 %, given that uncertainty ranges were not available in the literature. The resulting change in cost-effectiveness was then taken and the variables were ordered in a tornado diagram from the most to the least sensitive parameter.
viii) Key assumptions
  The model assumed that healthcare utilization is 100 % and constant for the entire population. Finally, our approach does not discriminate by rural–urban setting, assuming that all patients attend urban hospitals.

### Ethics

We did not submit this study for ethics committee review as no human subjects were involved in this research study. We used aggregated and de-identified secondary information from public domain sources.

## Results

Direct costs of prevention and treatment of diabetic foot ulcer in year 2012 varied depending on the different strategies (see Table 3). The total COI, using only direct costs for the three strategies adding preventive and treatment costs was US$74,470,803 for sub-optimal care, US$71,745,988 for standard care and US$96,800,267 for standard care plus temperature monitoring. Standard care strategy generates savings of roughly US$2*.*7 million compared to sub-optimal care. Switching to the standard care plus temperature monitoring strategy adds additional cost to the sub-optimal strategy of approximately US$22 million.

**Table 3:** Cost of illness (direct costs) attributed to diabetic foot (2012 US dollars)

Description	All strategies	*Sub-optimal care*		*Standard care*		*Standard care plus temperature monitoring*	
*Prevention strategy*							
Cost per person			US$65		US$185		US$406
Total people			206,298		206,298		206,298
Total cost of prevention			US$13,350,763		US$38,129,966		US$83,849,832
*Complications*	Cost/person	No. of people	Total cost	No. of people	Total cost	No. of people	Total cost
Hospital							
Healing with debridement	US$1,022	5416	US$5,535,166	2979	US$3,044,341	1148	US$1,172,820
Healing with major amputation	US$7,360	3658	US$26,921,264	2012	US$14,806,695	775	US$5,704,219
Healing with minor amputation	US$5,153	2897	US$14,930,136	1594	US$8,211,575	614	US$3,163,476
Death with major amputation	US$7,360	981	US$7,216,590	539	US$3,969,125	208	US$1,529,089
Death with minor amputation	US$5,153	777	US$4,002,215	427	US$2,201,218	165	US$848,010
*Subtotal*	*US$26,048*	*13728*	*US$58,605,371*	*7551*	*US$32,232,954*	*2909*	*US$12,417,613*
Outpatient							
Healing with outpatient visit	US$79	32032	US$2,514,669	17618	US$1,383,068	6787	US$532,821
Total No. of people/Total cost of treatment		*45761*	US$61,120,040	*25168*	US$33,616,022	*9696*	US$12,950,435
Total cost			US$74,470,803		US$71,745,988		US$96,800,267

**Fig. 2:**
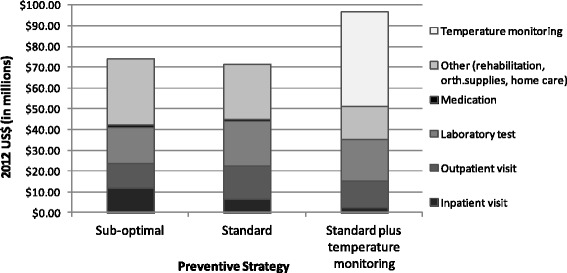
Resource usage for treatment and prevention of diabetic foot and related complications

Figure 2 shows the COI results by category of resources used. For sub-optimal and standard care, the major cost drivers are inpatient visits, outpatient visits, laboratory tests and other supplies. With standard care plus temperature monitoring, these costs are greatly reduced due to a larger reduction in the number of ulcerations, but the high cost of the thermometer device plays a significant role.

Table 4 provides the total outcomes averted and cost-effectiveness estimates for the strategies. In the baseline sub-optimal strategy, our model gives that there will be 1,757 deaths resulting from foot ulceration (using a 22*.*18 % prevalence rate). The standard care will avert 791 of these deaths (using a 12*.*2 % prevalence rate) and the standard care plus temperature monitoring strategy will avert 1,385 of the total deaths (using a 4*.*7 % prevalence rate). In terms of the health system burden, the standard care strategy will avert 2,087 major amputations compared to the sub-optimal care strategy, and standard care plus temperature monitoring will prevent 3,656 major amputations compared to the sub-optimal care strategy (see Table 4).

**Table 4:** Outcomes and cost-effectiveness of secondary prevention for diabetic foot

Comparison	Standard care vs. sub-optimal care	Standard care plus temperature monitoring vs. sub-optimal care	Standard care plus temperature monitoring vs. standard care
Differences in costs (∆ costs)			
Direct costs	-US$2,724,815	US$22,329,464	US$25,054,279
Direct costs + Indirect costs	-US$8,037,824	US$13,024,440	US$21,062,264
Differences in deaths (∆ deaths)	791	1,385	594
Differences in major amputations	2,087	3,656	1,568
Incremental cost-effectiveness ratio (∆ costs/∆ deaths)			
Direct costs	Cost-saving	ICER = 16,124	ICER = 42,169
Direct costs + Indirect costs	Cost-saving	ICER = 9,405	ICER = 35,450

The ICER for standard care versus the sub-optimal care is negative because of the cost-savings and is thus a highly favored outcome. The ICER for standard care plus temperature monitoring versus sub-optimal care indicates that US$16,124 dollars need to be spent for each additional death averted. The estimated indirect cost was US$6,719, which is the net value of the lost earnings for the next two years. When adding the indirect costs, the ICER for standard care plus temperature monitoring versus sub-optimal care becomes even more favorable, at US$9,405 per death averted. When comparing standard care plus temperature monitoring versus standard care, the ICER is over US$35,000 per death averted.

**Fig. 3:**
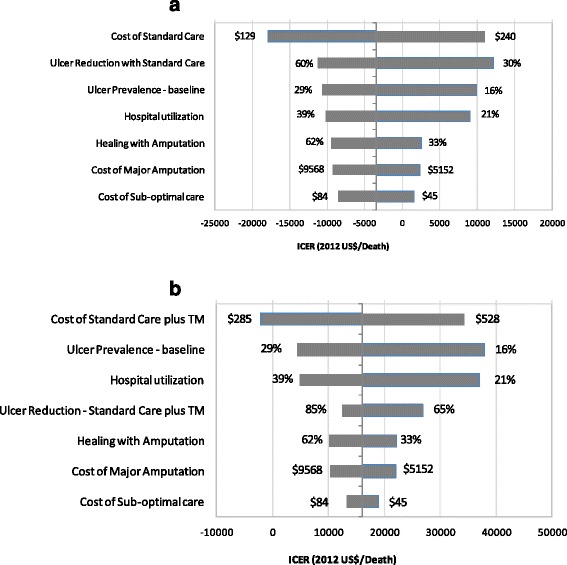
**a** Tornado diagram for standard care vs. sub-optimal care. **b** Tornado diagram for standard care plus temperature monitoring vs. sub-optimal care

At the current baseline estimate, the ICERs are either cost-saving or around US$16,000 per death averted for standard care and standard care plus temperature monitoring compared to sub-optimal care. Figure  3a and b show the results of a deterministic sensitivity analysis for these findings. The most sensitive variable in each case is the cost of the preventative strategy, which at a higher value, makes the ICER of standard care and standard care plus temperature monitoring less favorable. For the standard care option, the effectiveness of ulcer reduction by standard care, baseline ulcer prevalence and hospital utilization are the next most important variables. For standard care plus temperature monitoring, the second and third most sensitive parameters are baseline ulcer prevalence and hospital utilization, while the effectiveness of ulcer reduction was the fourth most sensitive variable.

## Discussion

### Main findings

Diabetic foot prevention and management certainly result in high economic burden for the Peruvian health system and society as well. The overall COI for prevention and complications management with standard care strategy would amount to a total savings of roughly US$2*.*7 million and avert 791 deaths. Our findings suggest that secondary prevention strategies are cost-effective for the individuals and the government of Peru if properly implemented. We note that ICER values per disability adjusted life year (DALY) are usually compared with three times the Gross Domestic Product (GDP) per capita of a country, which for Peru was US$6,568 in 2012 [20]. Our ICER using deaths averted is potentially more conservative given that disability benefits are not accounted for. ICER fell under three times the GDP per capita (US$16,124), we are confident it would be considered cost-effective [21].

Furthermore, standard care alone is even cost-saving, i.e. total costs decrease and deaths averted increase in comparison to sub-optimal care. When adding the indirect costs of lost productivity, the ICER for standard care versus sub-optimal care remains cost-saving and the standard care plus temperature monitoring versus sub-optimal care becomes more favorable, at US$9,405 per death averted. The sensitivity analysis showed that for the standard care only, the ICERs still fell under three times the GDP after varying the key parameters by +/−30 %.

From this analysis, a preventative strategy such as standard care not only improves the current level of care, but also leads to economic benefits in terms of societal cost-savings. Since prevention must be provided to a large number of patients at high-risk of ulceration, total costs of the prevention strategies are high; however, in the case of standard care plus temperature monitoring, the costs of thermometers would decline over time after the initial investment. For this strategy, even if the cost of the thermometer was zero, the strategy of standard care plus temperature monitoring would be cost-effective but not cost-saving. In the future, temperature monitoring technology may be integrated with electronic and mobile health prevention platforms that could further reduce the costs of personnel and phone services and make the intervention even more attractive.

### Comparison with the literature

Direct annual costs per patient calculated for management of foot ulcers and other diabetic foot complications are lower in Peru than costs estimated in other studies for developed countries [22–25]. Ulcers that require amputation can cost, per admission, from US$15,790 [26, 27] to as high as US$45,870 [23, 27]. In contrast, a study in Brazil [16] used a hypothetical cohort including all the Brazilian citizens with Type 2 diabetes and estimated a total annual cost of hospital admissions per patient starting from US$2,151.

The reported costs of diabetic foot prevention and treatment in developing and developed nations are difficult to compare, further emphasizing the importance of studies such as ours. Previous studies in cost-effective interventions to prevent and control diabetes agree with our estimates that comprehensive and multidisciplinary foot care leads to cost-savings [28–33]. One systematic review [28] included two studies that dealt with diabetic foot prevention, and one of these studies used an optimal care strategy similar to our standard care strategy [32]. An intensive prevention strategy including patient education, foot care and footwear was found to be cost-saving if the risk for foot ulcers and lower extremity amputations were reduced by 25 % [32] among those patients at risk of ulceration. In our study we assumed a reduction of ulceration rate of 45 % in line with lower bound estimates shown by the Centers for Disease Control and Prevention for potential reductions in amputation as a result of comprehensive foot care program [14, 15]. A retrospective cohort study from Austria using a Markov model, compared a dedicated screening program with conventional preventive care and concluded that the screening program would reduce costs by 29*.*8 % for mild ulcers and by 49*.*7 % for severe ulcers, primarily due to lower amputation rates [34]. Ollendorf et al. [35] estimated an increasing economic benefit associated to educational interventions, multidisciplinary teams, and the therapeutic shoe coverage. They did not, however, include the costs of the underlying intervention strategies.

Recent published studies indicate that the use of temperature monitoring is an effective way to prevent diabetic foot ulceration; however the cost-effectiveness of temperature monitoring had not been yet studied [29, 30]. Our study provides relevant findings about the potential of standard care plus temperature monitoring as a cost-effective prevention strategy even in a short-term horizon. However, standard care as recommended by the IDF is still more cost-effective and provides net savings for the society.

### Limitations

This study was performed from a societal perspective considering all direct healthcare costs, regardless of who paid; however, we did not include more assumptions about travel costs and waiting time given the lack of reliable information for those estimates. In this study, we tried to be as conservative as possible when considering the most appropriate health outcome indicator or whether to include indirect costs into the analysis. We have only considered productivity losses as indirect cost calculated under the "human capital" approach. This approach generates large estimates, but we presented our results of the ICER both including and excluding the productivity costs. For the purpose of this cost-effectiveness study, we chose two tangible outcomes as health effect indicators: amputations and deaths. Further cost-utility analysis may extend this to looking at a summary health outcome that incorporates morbidity such as the quality adjusted life year (QALY) or DALY. The estimates in this study are also limited by the lack of available data on clinical outcomes of preventative strategies specific to diabetic foot in Peru. As a result, we at times used secondary information from other country settings while aiming to use data from developing countries.

Another limitation is the model assumption that healthcare utilization was 100 % and constant for the entire population. We considered this assumption given the severe condition of these kinds of patients and given the lack of information on this issue. However, we expect that a lower utilization rate would lead to more major complications and more fatal outcomes of those who do not receive timely medical care.

We also assume that the parameters apply for the whole country and do not make a distinction between rural and urban populations. It is likely that this decision contributes to an underestimation of the total COI because the rural population will most likely incur larger indirect costs due to travel and time to reach a hospital in urban area. Our study calculated an ulcer rate using the data from a clinical study that occurred over an 18-month period [9], because longitudinal data is not yet available.

We only investigated the secondary prevention methods for diabetic foot, and given the lack of data for the burden of Type 1 diabetes, we did not include a comprehensive overhaul of diabetes monitoring and prevention in order to not overestimate and attribute costs that are also related with other complications (retinopathy, kidney diseases, among others). We chose to provide a detailed breakdown of costs, yearly costs, and unit costs, each presented separately. To ensure the wide applicability of our study, we have offered a breakdown of the likely routes for resource allocation in a given year. We are aware that there are intangible benefits for the patients and their families that are impossible to measure in monetary terms. We also only looked at the economic burden of the disease for the first year assuming that each prevention strategy achieved full scale-up.

While aiming to produce a conservative estimate of the cost of ulceration and amputation, we left out the added risk for recurrent ulcers and new amputations, which could be included in the future development of a probabilistic Markov model.

### Implications and further research

Diabetes prevalence in Peru is rising [36] and there is a low level of disease awareness and control [7]. Thus monitoring and treatment protocols are urgently needed. The health and economic impacts of diabetic foot are exacerbated in Peru because of insufficient preventative measures. We recommend validating costs and collecting better epidemiological data for diabetic foot disease in Peru.

## Conclusion

We have provided an economic evaluation for tracing costs related to prevention methods of diabetic foot disease in a developing country. Our study found a cost-saving result and decreased mortality associated with adherence to the standard care as recommended by the IDF relative to the current sub-optimal care that is provided in Peru. Additionally, we found that standard care plus temperature monitoring was a promising cost-effective strategy and could be even more cost-effective with declining price in the thermometer device. Our results highlight the important economic impact on savings and lives saved that diabetic foot programs can yield.

## Additional file

Additional file 1:
**Cost estimates of procedures and preventative strategies using the** **ingredients-based** **approach (Peru, 2012).** (XLS 57 kb)

## Abbreviations

COI: Cost-of-illness
GDP: Gross domestic product
ICER: Incremental cost-effectiveness ratio
IDF: International Diabetes Federation
PEN: Peruvian Nuevos Soles
